# Meibomian gland morphological changes in ocular herpes zoster patients based on AI analysis

**DOI:** 10.3389/fcell.2022.1094044

**Published:** 2022-12-01

**Authors:** Xinxin Yu, Xu Jia, Zuhui Zhang, Yana Fu, Jing Zhai, Naimei Chen, Qixin Cao, Zhentao Zhu, Qi Dai

**Affiliations:** ^1^ School of Ophthalmology and Optometry, The Eye Hospital of Wenzhou Medical University, Wenzhou, China; ^2^ Department of Ophthalmology, Huaian Hospital of Huaian City, Huaian, China; ^3^ Huzhou Traditional Chinese Medicine Hospital Affiliated to Zhejiang University of Traditional Chinese Medicine, Huzhou, China

**Keywords:** varicella-zoster virus, herpes zoster ophthalmicus, artificial intelligence, convolutional neural network, meibomian gland morphology

## Abstract

Varicella-zoster virus (VZV) infections result in a series of ophthalmic complications. Clinically, we also discover that the proportion of dry eye symptoms was significantly higher in patients with herpes zoster ophthalmicus (HZO) than in healthy individuals. Meibomian gland dysfunction (MGD) is one of the main reasons for dry eye. Therefore, we hypothesize that HZO may associate with MGD, affecting the morphology of meibomian gland (MG) because of immune response and inflammation. The purpose of this study is to retrospectively analyze the effect of HZO with craniofacial herpes zoster on dry eye and MG morphology based on an Artificial intelligence (AI) MG morphology analytic system. In this study, 26 patients were diagnosed as HZO based on a history of craniofacial herpes zoster accompanied by abnormal ocular signs. We found that the average height of all MGs of the upper eyelid and both eyelids were significantly lower in the research group than in the normal control group (*p* < 0.05 for all). The average width and tortuosity of all MGs for both upper and lower eyelids were not significantly different between the two groups. The MG density of the upper eyelid and both eyelids were significantly lower in the HZO group than in the normal control group (*p* = 0.020 and *p* = 0.022). Therefore, HZO may lead to dry eye, coupled with the morphological changes of MGs, mainly including a reduction in MG density and height. Moreover, it is important to control HZO early and timely, which could prevent potential long-term severe ocular surface injury.

## Introduction

Herpes zoster (HZ) is caused by the reactivation of the latent varicella-zoster virus (VZV) within the sensory ganglia. VZV is a member of the Herpesviridae family that affects sensory neurons following a varicella infection in childhood (chickenpox) (Jeng, 2018). VZV tends to be reactivated when immunosuppression is caused by medication, illness, or advanced age. Different degrees of eye lesions can occur when the virus invades the ocular branch of the trigeminal nerve. VZV infections result in a series of ophthalmic complications, including involvement of the skin and cornea, as well as the iris, retina, optic nerve, and other cranial nerves. An estimated 10%–20% of people with HZ will develop herpes zoster ophthalmicus (HZO) (Johnson et al., 2015), such as ptosis, blepharitis, keratitis, scleritis, uveitis, glaucoma, diffuse or focal choroiditis, and acute retinal necrosis (Davis and Sheppard, 2019; Niederer et al., 2021). These manifestations are considered to be related to active infection as well as to the host’s immune response and inflammation. In addition, HZO is chronic and recurrent, which is different from cutaneous herpes zoster mostly occurs only once in a lifetime.

Clinically, we also found that the proportion of dry eye symptoms, such as dryness, foreign body sensation, redness, and burning, was significantly higher in patients with HZO than in healthy individuals. A case-control study confirmed that patients with a history of herpes simplex (HS) keratitis often experience ocular dryness (Simard-Lebrun et al., 2010; Jabbarvand et al., 2015; Roozbahani and Hammersmith, 2018). We consider that the underlying mechanisms of dry eye owing to HZ infection are the same as those of HS infection, including corneal nerve function abnormalities, chronic inflammation of the ocular surface, and so on.

The Tear Film and Ocular Surface Dry Eye Workshop II in 2017 defined dry eye disease (DED) as a multifactorial disease of the ocular surface characterized by a loss of homeostasis of the tear film and accompanied by ocular symptoms. Tear film instability and hyperosmolarity, ocular surface inflammation and damage, and neurosensory abnormalities play etiological roles in DED (Craig et al., 2017). Evaporative dry eye accounts for the majority of dry eye and is most often caused by meibomian gland dysfunction (MGD). The meibomian gland (MG) is an important sebaceous gland located in the eyelid that secretes lipids, an important component of the tear film (Yu et al., 2016; Stapleton et al., 2017). Abnormalities in the quantity and quality of lipids can damage the lipid layer of the tear film, severely affecting the physicochemical properties of the tear film and reducing its stability, which can lead to evaporative dry eye (Bron et al., 2004; Yeotikar et al., 2016). The function of MG is closely related to its morphology. Studies have shown that the morphology of MG is a sensitive early diagnostic indicator of MGD (Yeotikar et al., 2016; Adil et al., 2019). We hypothesize that HZO may be associated with MGD, similar to many inflammatory diseases, such as Sjögren syndrome, rheumatoid arthritis, and rosacea (Wang et al., 2018), affecting the morphology of MGs because of immune response and inflammation.

Artificial intelligence (AI) analytic system based on a convolutional neural network (CNN) is a relatively new technology in the field of computer vision. CNN is a feedforward neural network that can reduce manual analysis errors and save time when used to manipulate MG images (Rajkomar et al., 2017; Chen et al., 2018a; Deng et al., 2021). Our previous study confirmed that a CNN-based AI system could be used to analyze MG morphological characteristics efficiently and effectively (Zhang et al., 2022). Therefore, the purpose of this study was to retrospectively analyze the effect of HZO with craniofacial herpes zoster on dry eye and MG morphology based on AI MG morphology analytic system. To the best of our knowledge, this is the first study to investigate the change of MGs morphology after craniofacial herpes zoster.

## Materials and methods

### Subjects

We conducted a retrospective, self-control study at the Affiliated Eye Hospital of Wenzhou Medical University from December 2018 to May 2021. This study was approved by the Research Ethics Committee of the Eye Hospital, Wenzhou Medical University. All the procedures adhered to the tenets of the Declaration of Helsinki. Informed consent to publish was obtained from all participants before including them in the study.

The patients were diagnosed as HZO based on a history of craniofacial herpes zoster accompanied by herpes zoster keratitis. Participants wearing contact lenses, with chalazion, active nasolacrimal infections, severe systemic diseases, and a history of ocular trauma or surgery were excluded from the study. Basic information was collected, including patients’ age at diagnosis of craniofacial herpes zoster, patient age at diagnosis of HZO, interval time from diagnosis of craniofacial herpes zoster to the diagnosis of HZO, duration of follow-up, HZO eye, and gender. The HZO eye was used as the research group, whereas, the contralateral eye was used as the control group**.**

### AI model and morphology analysis

**FIGURE 1:**
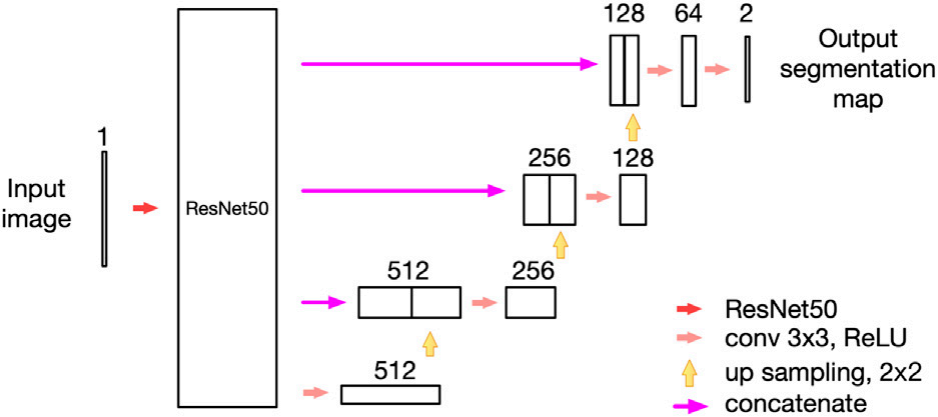
The network structure of the ResNet50_U-net model in this study.

A Keratograph 5M (K5M; Oculus, Wetzlar, Germany) was used to perform meibography scans of the upper and lower eyelids of both eyes. An AI analytical system for MG morphology was used to automatically analyze and calculate the morphological parameters of MGs. The AI model used in this study was a revision of our previously published model (Figure 1) (Zhang et al., 2022). We replaced the 50-layer ResNet (ResNet50) with the max-pooling layers of the U-net model, however, the up-sampling layer remained the same. We called it ResNet50_U-net model, and the AI system achieved 92% accuracy (IoU) and 100% repeatability in MG segmentation for both upper and lower eyelids.

1) Vagueness value (Yin and Gong, 2019)

MGs display intensity in meibography. Firstly, grayscale values of each pixel of meibography were measured by a computer. Secondly, the algorithm of the vagueness value is as follows:

Average grayscale value of MGs = Total grayscale values of MGs/Total pixels of MGs
Average grayscale value of background = (Total grayscale values of tarsus−Total grayscale values of MGs)/(Total pixels of tarsus−Total pixels of MGs)
Vagueness value = Average grayscale value of MGs−Average grayscale value of background

**FIGURE 2:**
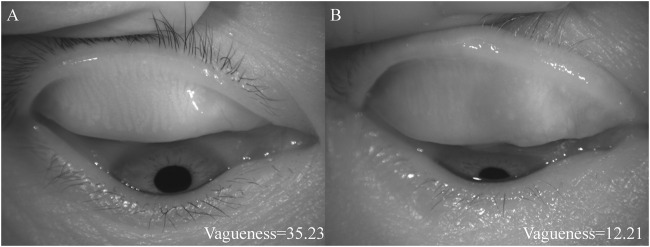
The diagrams of different vagueness values. **(A)** showed the higher vagueness value, **(B)** showed the lower vagueness value.

Figure 2 shows the different vagueness values. The higher the value, the clearer the picture.

2) MG morphological indexes

The morphological parameters of MGs included height, width, tortuosity, and density. MG height was the vertical difference between the top and bottom pixels of the MG, and MG width is the area divided by height. The MG density was defined as the ratio of the sum of the area of the MGs to the total area of the tarsus, as follows:

MG density = Sum pixels of all MGs/Total pixels of the tarsus

We defined MG tortuosity as the ratio of the imaginary straight length between the two nodes and the actual length of each MG. The MG perimeter was a pixel at the edge of the MG. Because the outlines of the MGs were irregular, some of them MGs were tilted. Therefore, we used the minimum external rectangle to frame the outline of the MGs and calculate their height.

MG tortuosity = MG perimeter/(2 × height of the minimum external rectangle of the MG)−1

### Statistical analysis

Statistical analysis was performed using IBM SPSS 26.0 statistical software. Sample characteristics were summarized using descriptive statistics including means and SD for continuous measures, and frequencies and percentages for categorical measures. The normality of all datasets was tested using the Kolmogorov-Smirnov test. The paired *t*-test or the Wilcoxon signed-rank test was used to compare the differences between HZO eyes and normal eyes. A *p* value < 0.05 was considered significant.

## Results

**FIGURE 3:**
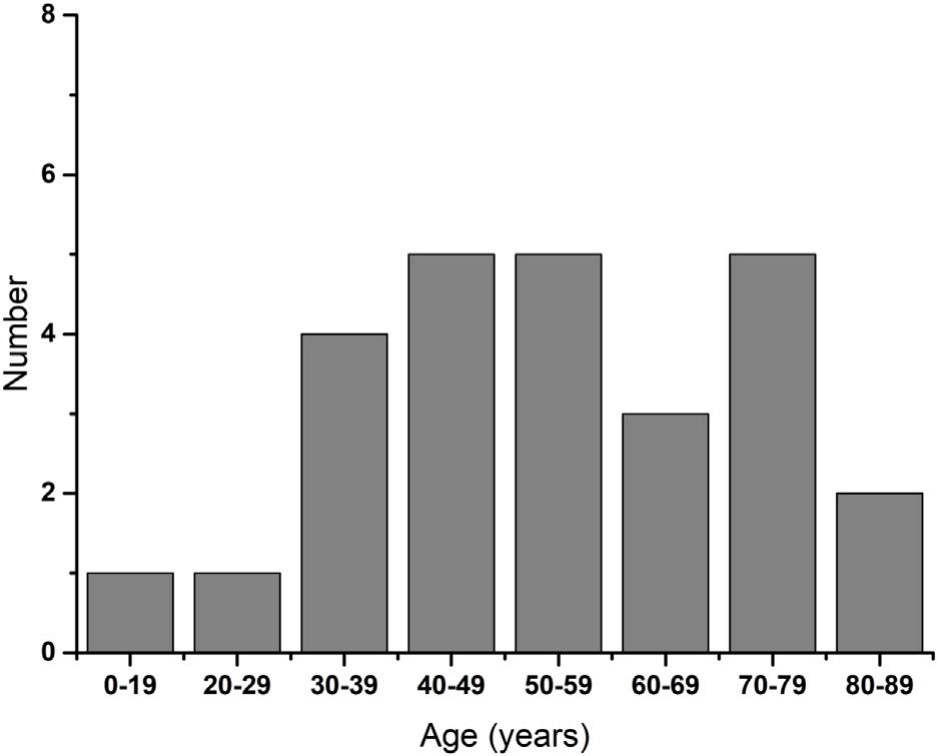
Distribution of age at the time of diagnosis of HZO by decade.

This study included 26 patients who had been diagnosed with HZO. The demographic features were presented in Table 1. The mean age was 54.16 ± 18.59 years when diagnosed as HZO and the majority was male (76.9%). Age stratification into 10-year increments revealed that HZO could occur at any age and increase with age (Figure 3). The mean interval time from craniofacial herpes zoster to HZO was 6.50 months. The tear break-up time (TBUT) and tear meniscus height (TMH) were not statistically different between the research group and the control group.

**TABLE 1:** Demographic features and baseline characteristics.

Parameters	
Age at diagnosis of HZO (years, mean ± SD)	54.16 ± 18.59
Gender (n, male/female)	20/6
Eye involved (n, OD/OS)	9/17
Interval time from diagnosis of craniofacial herpes zoster to a diagnosis of HZO (months)	6.50
	(3.00, 12.25)
TBUT (seconds)	
Research group	5.76 ± 2.86
Control group	7.68 ± 4.92
TMH (mm)	
Research group	0.22 (0.18, 0.26)
Control group	0.19 (0.16, 0.23)

HZO, herpes zoster ophthalmicus; TBUT, tear break-up time; TMH, tear meniscus height.

**FIGURE 4:**
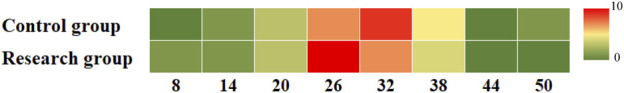
The heat map of vagueness distribution in the two groups.

Table 2 showed the MG vagueness value in the research group and normal control group. The vagueness value of the upper eyelid was lower in the research group (27.24 ± 10.32) than in the normal control group (30.54 ± 11.51), but there was no statistical difference (*p* > 0.05). The results of the lower eyelid and both eyelids were consistent with those of the upper eyelid. Figure 4 shows the heat map of vagueness distribution in the two groups.

**TABLE 2:** MG vagueness value in the research group and normal control group.

	Research group	Control group	*p* value
Vagueness value of the upper eyelid	27.24 ± 10.32	30.54 ± 11.51	0.208
Vagueness value of the lower eyelid	22.76 ± 10.18	23.90 ± 11.92	0.693
Vagueness value of both eyelids	25.00 ± 6.85	27.22 ± 7.49	0.270

To understand the effect of VZV infection on MG morphology, we calculated the average height, width, tortuosity, and density of all MGs. The results were presented in Table 3. The average height of all the MGs of the upper eyelid and both eyelids were significantly lower in the research group than in the normal control group (*p* < 0.05 for all). However, the average height of all MGs of the lower eyelid in the research group (87.52 ± 19.13) was not significantly lower than in the control group (89.90 ± 19.38; *p* = 0.603). Except for the average width of all MGs of both eyelids, the average width of all MGs and the average tortuosity of all MGs of the upper eyelid, lower eyelid, and both eyelids were not significantly different between the two groups. The MG density of the upper eyelid and both eyelids was significantly lower in the research group than in the normal control group (*p* = 0.020 and *p* = 0.022, respectively).

**TABLE 3:** MG parameters of the two study groups.

	Parameters	Research group	Control group	*p* value
Upper eyelid	Average height of all mgs	126.46 ± 43.81	149.92 ± 41.44	0.014
	Average width of all mgs	21.26 ± 4.76	24.76 ± 8.78	0.058
	Average tortuosity of all mgs	0.43 ± 0.10	0.50 ± 0.23	0.485
	MG density	0.19 ± 0.08	0.22 ± 0.08	0.020
Lower eyelid	Average height of all mgs	87.52 ± 19.13	89.90 ± 19.38	0.603
	Average of all mgs	26.50 ± 5.89	28.00 ± 5.25	0.338
	Average tortuosity of all mgs	0.53 ± 0.15	0.58 ± 0.14	0.174
	MG density	0.16 ± 0.07	0.19 ± 0.08	0.088
Both eyelids	Average height of all mgs	107.07 ± 26.58	119.91 ± 25.76	0.038
	Average width of all mgs	23.93 ± 4.58	26.39 ± 5.43	0.039
	Average tortuosity of all mgs	0.48 ± 0.10	0.52 ± 0.12	0.139
	MG density	0.17 ± 0.06	0.21 ± 0.06	0.022

**FIGURE 5:**
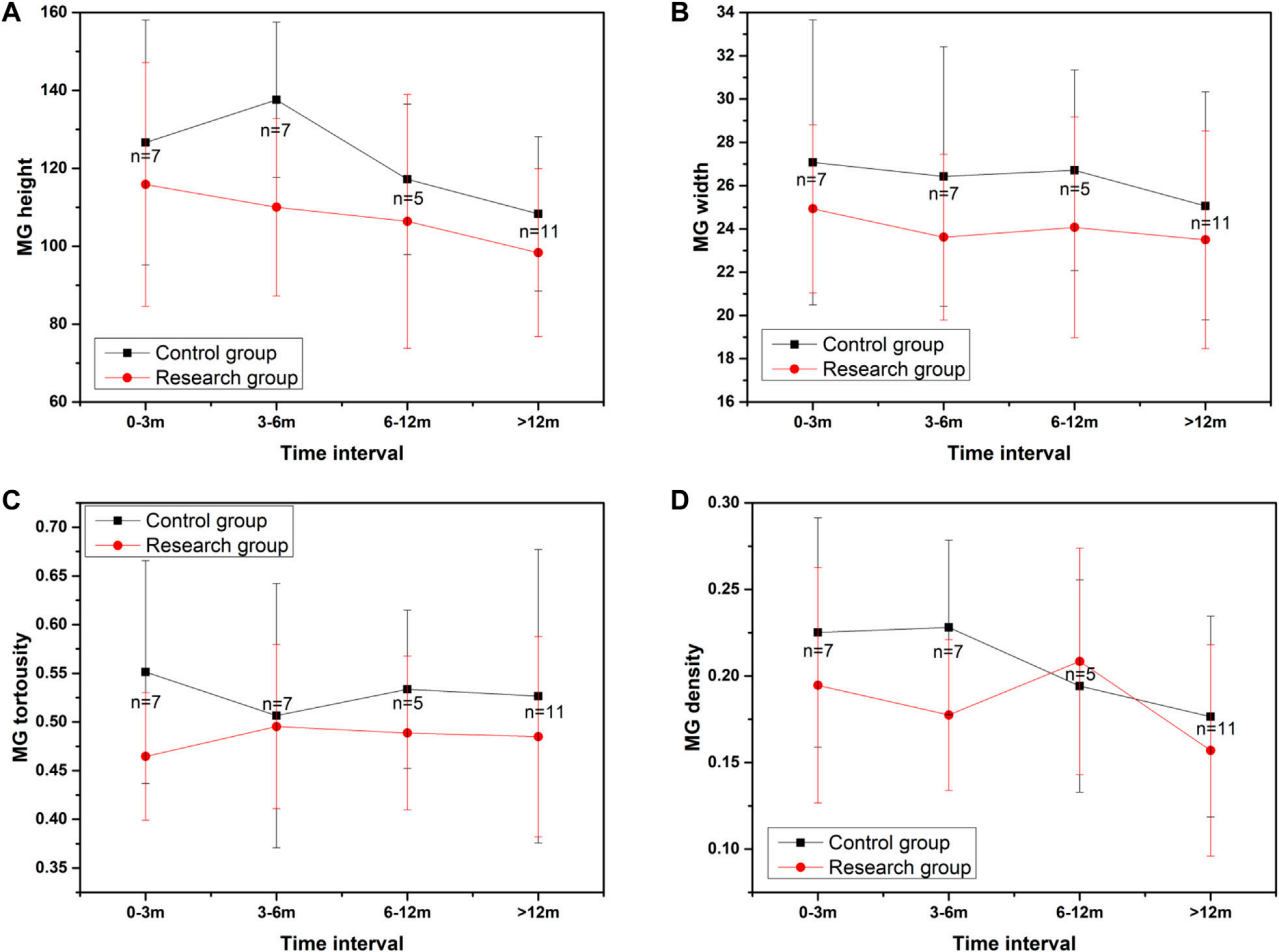
The changes in MG morphology parameters over time. **(A)** showed the changes in the average height of all MGs during follow-up visits, **(B)** showed the changes in the average width of all MGs during follow-up visits, **(C)** showed the changes in the average tortuosity of all MGs during follow-up visits, **(D)** showed the changes of MG density during follow-up visits.

We also compared the changes in the average height of all MGs, the average width of all MGs, the average tortuosity of all MGs, and MG density between the two groups during follow-up visits (Figure 5). The results showed that the parameters of MG morphology, such as the average height of all MGs, the average width of all MGs, the average tortuosity of all MGs, and MG density, decreased over time. Although there was no significant difference between the research group and the control group for parameters of MG morphology over time, the parameters of MG morphology in the research group were lower than those in the normal control group as time went on. The results were shown in Figure 5 and Table 4.

**TABLE 4:** The changes in MG morphology parameters over time.

Parameters	Time interval	Research group	Control group	*p* value
MG height	0–3 min	115.85 ± 31.32	126.62 ± 31.41	0.270
	3–6 min	110.02 ± 22.78	137.60 ± 19.91	0.058
	6–12 min	106.39 ± 32.61	117.20 ± 19.31	0.344
	>12 min	98.34 ± 21.53	108.31 ± 19.81	0.347
MG width	0–3 min	24.93 ± 3.88	27.07 ± 6.58	0.247
	3–6 min	23.62 ± 3.83	26.43 ± 6.00	0.280
	6–12 min	24.07 ± 5.10	26.71 ± 4.63	0.343
	>12 min	23.50 ± 5.03	25.06 ± 5.27	0.484
MG tortuosity	0–3 min	0.46 ± 0.07	0.55 ± 0.11	0.075
	3–6 min	0.50 ± 0.08	0.51 ± 0.14	0.870
	6–12 min	0.49 ± 0.08	0.53 ± 0.08	0.333
	>12 min	0.48 ± 0.10	0.53 ± 0.15	0.439
MG density	0–3 min	0.19 ± 0.07	0.23 ± 0.07	0.067
	3–6 min	0.18 ± 0.04	0.23 ± 0.05	0.108
	6–12 min	0.21 ± 0.07	0.19 ± 0.06	0.666
	>12 min	0.16 ± 0.06	0.18 ± 0.06	0.383

The MG morphology parameters, including the average height, width, tortuosity, and MG density, had no significant correlations with TBUT and TMH in either the research group or the control group (*p* > 0.05 for all).

**FIGURE 6:**
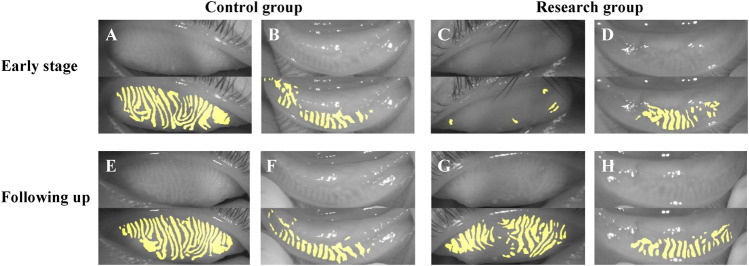
The follow-up results of a child. **(A–D)** showed the photograph of MG in the early stage of HZO, and **(E–H)** showed the photographs of MG following 2 years after HZ infection. **(A,B,E,F)** were the control eyes without HZ infection, and **(C,D,G,H)** were the research eyes with HZ infection.

Figure 6 showed the follow-up results of a child. We investigated the photograph of MG in the early stage of HZO and 2 years after infection. In the early stage, the photograph of MG in the eye with HZO was blurry. However, the photograph of MG became clear when the inflammation was under control. However, it was still more twisted and fragmented than the other eye.

**FIGURE 7:**
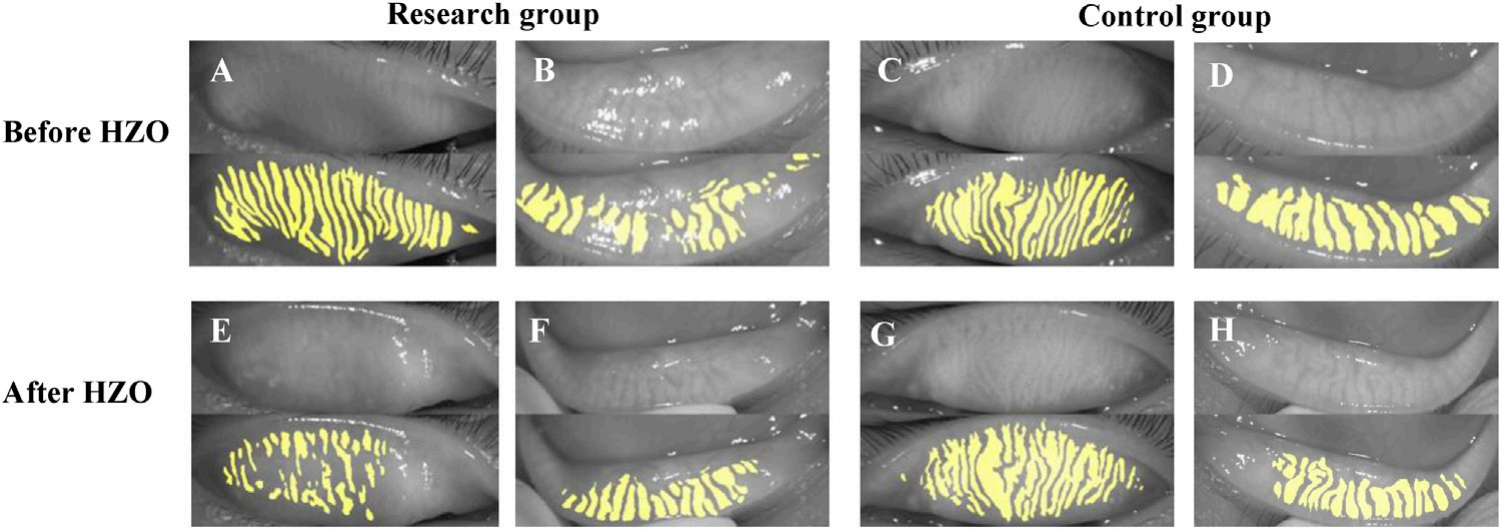
The photograph of MG before and after HZO. **(A–D)** showed the photograph of MG before HZO, and **(E–H)** showed the photograph of MG after HZO. **(A,B,E,F)** were the research eyes with HZ infection, and **(C,D,G,H)** were the control eyes without HZ infection.

We also investigated the photographs of MG before and after HZO (Figure 7). There was no significant difference between the sides before HZO for the photograph of MG. The MG after HZO became more twisted, and swelled with more fragmentation than the status before HZO.

## Discussion

Ocular inflammation may cause typical ocular surface changes in patients with HZO and craniofacial herpes zoster. Additionally, the nerve damage inherent to VZV infection results in a neurotrophic keratopathy with diminished corneal sensation, loss of corneal epithelial integrity, and tear dysfunction (Liesegang, 2008; Ghaznawi et al., 2011). Many studies have shown a strong association between MGD and ocular surface inflammation (Wang et al., 2018). Mathers et al. reported that patients with chronic inflammation, such as blepharitis and conjunctivitis, experienced a greater loss of MGs (Mathers et al., 1991; Mathers and Billborough, 1992). Thus, we hypothesized that HZ infection associated ocular surface inflammation might cause peri-glandular inflammation and subsequent MG loss.

MG atrophy causes abnormalities in the quantity and quality of lipids, which can damage the lipid layer of the tear film and lead to MGD. A detailed analysis of MGs morphology is important to determine the extent and severity of MGD (Deng et al., 2021). Most AI-assisted morphologic studies of the MGs have focused on the MGs grading system, and few have quantitatively discussed the various morphological characteristics, such as tortuosity and density. The present study utilized an AI system based on a convolutional neural network, to automatically analyze MG morphology, including the height, width, tortuosity, density, and vagueness value of the MGs. Currently, studies on MG morphology have focused on the tortuosity of MGs, whereas studies on MG density are relatively rare. Xiao et al. (2019) found that meiboscore, gland distortion, and MG length had an excellent ability to differentiate between patients with MGD and healthy subjects. They considered that structural MG changes were closely associated with MGD progression. Shirakawa et al. (2013) compared MG morphology between children and adults and confirmed that MG density in the upper eyelid was significantly greater in children than in adults. Shirakawa et al. (2013) defined MG density as the number of MGs divided by eyelid width. In our study, MG density was calculated as the ratio of the sum of the area of the MGs to the total area of the tarsus, which is a continuous, objective, quantitative index that is more accurate than MG grading and avoids subjective errors. We compared the MG morphology (height, width, tortuosity, density, and vagueness value of the MGs) with and without HZO using this novel AI system.

Some morphological characteristics of MGs, such as length, width, and shape irregularity, have been suggested to be valuable for assessing MGD. We found the average height of all MGs and MG density in the upper eyelid and both eyelids were significantly decreased in the research group compared to the normal controls (Table 3). Similar to our study, some research groups had described other changes in MG morphology, such as MG thickness and length, in patients with dry eye. A related study found that the number of distorted glands and MG thickness, density, and length were inversely correlated with meibum expressibility (Xiao et al., 2019). However, in our study, the average width of all MGs and average tortuosity of all MGs of the upper eyelid, the lower eyelid, and both eyelids were not significantly different between the research group and the normal control group (Table 3). In our previous study, the early stage of MGD showed an increase in tortuosity, while in patients with more severe MGD, the change in tortuosity was no longer noticeable and the density decreased significantly (Lin et al., 2020). In the present study, we assumed that ocular inflammation caused by HZO was severe, thus leading to severe MGD associated MGs shortening and decreased MGs density, rather than increased MG tortuosity. Furthermore, conjunctival inflammation and edema could affect vagueness value by reducing transmission of infrared light. We also calculated the vagueness value in the research group and normal control group. The research group was lower than the normal control group, but there was no statistical difference. We speculated the cause that some patients had a certain period of time from the onset of the disease and the conjunctival inflammation had subsided when the meibographs were taken. This phenomenon was clearly observed in meibographs of some patients followed from the early stage of the disease.

In addition, we also compared the changes in MG morphology parameters over time, such as the average height of all MGs, the average width of all MGs, the average tortuosity of all MGs, and MG density. The results showed that the parameters of MG morphology were decreasing as time went on. Although there was no statistical difference regardless of the presence or absence of HZO, the parameters of MG morphology in the research group were always lower than those in the normal control at different periods. We considered the possible reason was the insufficient sample size for each period. We plan to include patients with ocular herpes simplex in further studies. The population of ocular herpes simplex patients similar to HZO is larger and more common.

What’s more, we observed two special cases (Figures 6, 7). Figure 6 showed the follow-up results for a child. In the early stages of HZO, the photograph of MG on the side of the HZO was blurry. However, the meibography images became clear when inflammation was controlled. This suggests that palpebral conjunctival edema caused by inflammation, leading to blurred meibography images and lower optical density also plays an important role in MG density decrease (Chen et al., 2018b). Figure 7 showed that there was no significant difference between the eye with HZO and the normal contralateral eye in the photograph of MG before the attack of craniofacial herpes zoster. After the onset of craniofacial herpes zoster, the MGs became twisted, swollen, and fragmented in the HZO eye. Comparing these two cases, we believe it is important to control HZO early. Early and timely control of inflammation could prevent potential complications, such as peri-glandular inflammation, loss of MGs, dry eye, and so on.

The present study has some limitations. The sample size was small and the age span of the patients was large, which might be the reason for the lack of statistically significant differences in parameters between the research group and the normal control group. The changes in MGs over time in HZO patients should be verified in a future study with a larger sample.

## Conclusion

In conclusion, similar to many ocular surface inflammatory diseases, HZO may lead to dry eye, and be accompanied by morphological changes of MGs, mainly including a reduction in MG density and height. Moreover, it is important to control HZO early and timely, which could prevent potential long-term severe ocular surface injury.

## Data Availability

The raw data supporting the conclusion of this article will be made available by the authors, without undue reservation.
